# Social Housing and Health in Manitoba

**DOI:** 10.23889/ijpds.v1i1.376

**Published:** 2017-04-19

**Authors:** Mark Smith, Leslie Roos, Greg Finlayson, Patricia Martens, Jim Dunn, Heather Prior, Carole Taylor, Ruth-Ann Soodeen, Aynslie Hinds, Charles Burchill, Wendy Guenette

**Affiliations:** 1 Manitoba Centre for Health Policy; 2 University of Manitoba; 3 McMaster University

## Objective

Fourteen years of social housing data (1995-2008) were acquired from the provincial government. This allowed for an unprecedented opportunity to describe the population of individuals living in social housing and, through data linkage, to compare them to the rest of the province on a number of health and social indicators.

## Method(s)

Using data from the entire population of the province of Manitoba, Canada, cross-sectional comparison were made between those living in social housing and those not on 19 indicators of morbidity, mortality, health care utilization and social development. Regression models were developed to control for age, sex, region of residence, comorbidities, income and neighborhood level SES.

## Results

50% of the population in social housing are under the age 20, 75% are female and 50% of applicants receive some form of income assistance. As expected there are significant differences on most health status measures when compared to individuals not in social housing. However, after controlling for confounding factors most differences between the two groups disappear indicating that there is no independent effect of living in social housing. A few exceptions were noted on measures of total respiratory morbidity, mammography and high school completion rates, the later showing a very significant interaction with neighborhood level SES.

## Conclusion

Despite overall poor health status, after controlling for income and other confounding factors individuals in social housing score no worse on many measures of health care utilization and prevention. High school completion rates, in particular, showed a very strong interaction with neighborhood level SES. Policy implications of this research are discussed.

